# Editorial: Emerging relevance of molecular profiling in global cancer research and management

**DOI:** 10.3389/fgene.2025.1679501

**Published:** 2025-08-28

**Authors:** Premila Leiphrakpam, Srijan Shukla, Chandrakanth Are

**Affiliations:** ^1^ Graduate Medical Education, College of Medicine, University of Nebraska Medical Center, Omaha, NE, United States; ^2^ Division of Surgical Oncology, Department of Surgery, College of Medicine, University of Nebraska Medical Center, Omaha, NE, United States; ^3^ Department of Surgical Oncology, Basavatarakam Indo-American Cancer Hospital and Research Institute, Hyderabad, India

**Keywords:** global cancer, tumor profiling, molecular mechanism, biomarker discovery, early diagnosis

Cancer remains a major global health burden, contributing significantly to morbidity and mortality worldwide (Kocarnik et al., 2022). In 2022 alone, an estimated 20.0 million new cases and 9.7 million cancer-related deaths were reported globally, and these numbers are projected to increase to 35 million new cases and 19 million deaths by 2050 (Bray et al., 2024).

In recent years, the field of oncology has increasingly shifted toward personalized medicine, largely driven by advances in tumor molecular profiling. This technique, which identifies specific biomarkers in tumor tissue or circulating blood, has revolutionized our understanding of the molecular drivers of cancer. Molecular profiling has refined the classification of cancer types and subtypes and enhanced diagnostic, prognostic, and therapeutic strategies. In many high-income countries, these innovations have become integrated into standard clinical care, allowing for treatments tailored to individual tumor characteristics. However, these scientific advancements have not translated into improved outcomes equally on a global scale. Significant disparities persist, particularly in low- and middle-income countries, where access to molecular testing and targeted therapies is limited. Bridging this gap requires sustained ongoing efforts to identify and validate novel cancer biomarkers and support the global implementation of molecular profiling in routine oncology practice.

This Research Topic in *Frontiers in Genetics* features five articles that highlight innovative translational research in biomarker discovery, each contributing to the broader goal of advancing personalized medicine in cancer care.

In the article by Song et al., the authors investigated the role of metabolic reprogramming, a well-established hallmark of cancer, by developing a metabolism-associated prognostic model for pancreatic cancer. Using weighted gene co-expression network analysis (WGCNA), they identified five key metabolic hub genes (*DLX3*, *HMGA2*, *SPRR1B*, *MYEOV*, and *FAM111B*) linked to distinct metabolic phenotypes and patient prognosis. The study also demonstrated strong correlations between these metabolism-associated factors and DNA damage repair (DDR) mechanisms, emphasizing the role of altered metabolism reprogramming in promoting malignant progression in pancreatic cancer. The authors suggested that these findings highlight the clinical potential of targeting tumor metabolism within precision oncology frameworks.

Our understanding of the molecular mechanisms driving cancer initiation, tumor heterogeneity, classification, and the development of personalized therapeutic strategies has significantly expanded with advances in cancer genomic analysis, yet gaps remain (Wang et al., 2023). In their article, Lozada-Martinez et al. conducted a bibliometric analysis to examine the landscape and evolution of cancer genomics research in Latin America, a region that has been historically underrepresented in this field. Using Scopus data from 1997 to 2023, they identified 1,534 cancer genomic publications by Latin American researchers and found that Brazil led the region in publication volume, researcher productivity, and both regional and international collaboration. The authors observed a shift in research focus over time from broad genomic studies to more specialized areas such as cancer stem cells and personalized medicine. The authors noted that these findings highlighted the need to strengthen research infrastructure and regional and global research networks, particularly in resource-limited settings. The article by Drobyshev et al. investigated the transcriptional landscape of telomerase reverse transcriptase (*TERT)*, the gene encoding the telomerase catalytic subunit, across 27 tumor types using RNA sequencing data from 1,039 cancer samples. *TERT* reactivation, often linked to poor prognosis, exhibited a bimodal pattern, with ∼27% of tumors classified as TERT-negative and the remaining as TERT-positive, and these tumor types had distinct molecular characteristics. Although ∼13% of TERT-positive cancers carried TERT promoter mutations (C228T or C250T), these mutations did not correspond with higher TERT expression, suggesting alternative regulatory mechanisms. TERT-positive cancers also showed reduced L1 retrotransposon expression, particularly in tumors with promoter mutations. Furthermore, TERT expression correlated with 17 known therapeutic target genes, highlighting its potential role in shaping treatment response and survival outcomes.

Multi-omics technologies, which integrate data from genomics, transcriptomics, proteomics, and metabolomics, have revolutionized cancer research by providing a comprehensive view of the molecular landscape of tumors, yet gaps remain (Menyhárt and Győrffy, 2021). In their article, He et al. conducted a multi-omics analysis to investigate primary, recurrent, and metastatic tumors across a pan-cancer patient cohort. This integrative approach identified distinct molecular mechanisms associated with early recurrence and metastasis. Notably, BPIFB1 overexpression and high B-cell infiltration were associated with early recurrence, while overexpression/amplification of ANKRD22 and LIPM, mutations in IGHA1 and MUC16, increased fibroblast infiltration, M1 macrophage polarization, and alterations in DNA repair mechanisms were linked to early metastasis. The authors noted that these findings offer a context-specific understanding of biomarkers associated with recurrence and metastasis with potential prognostic and therapeutic relevance. In the article by Sicheng et al., the authors investigated the potential causal relationship between atrial fibrillation (AF) and gastric cancer using a two-sample Mendelian randomization approach. By analyzing genome-wide association study (GWAS) data and constructing a genetic instrument comprising 136 AF-associated single-nucleotide polymorphisms (SNPs), the authors identified a negative association between AF and gastric cancer risk, independent of known intermediary factors such as chronic gastritis, *Helicobacter pylori* infection, and alcohol consumption. They also identified 62 shared genes between AF and gastric cancer, many of which are implicated in cardiovascular disease, inflammation, and tumorigenesis, suggesting complex biological interconnections. These findings provide novel insights into the association of AF and gastric cancer and may inform future research on the pathogenesis and potential therapeutic strategies for gastric cancer.

The Research Topic highlights the growing potential of biomarker discovery in advancing personalized cancer care. By uncovering novel molecular insights and offering innovative diagnostic and prognostic capabilities, this body of research contributes to the evolution of precision oncology. However, to realize the full potential of precision oncology on a global scale, continued research and clinical validation must be paired with efforts to expand equitable access to molecular diagnostics and targeted therapies globally, especially in under-resourced regions.
